# Supplementary material for: Microfluidic control over topological states in channel-confined nematic flows
Source: Nat Commun. 2020 Jan 2;11:59. doi: 10.1038/s41467-019-13789-9 (PMC6940393; doi:10.1038/s41467-019-13789-9)
Supplement: Supplementary file 1 — Supplementary Information [file 41467_2019_13789_MOESM1_ESM.pdf]

Electronic Supplementary Material

Microfluidic control over topological states in channel-confined nematic flows

Čopar *et al.*

Supplementary Figures

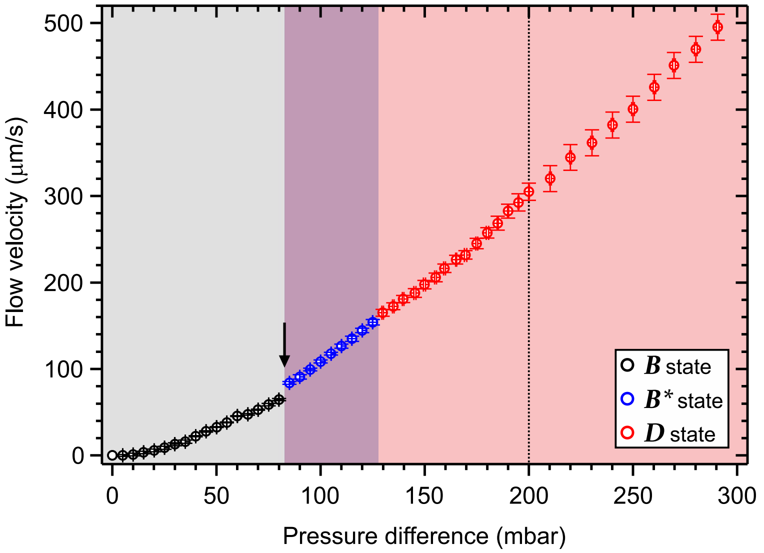

Supplementary Figure 1: **Flow velocity as a function of the stationary pressure difference.** The pressure difference in 100 µm wide and 12 µm deep homeotropic channel was varied by pressure control system with ±0.05 mbar precision up to 200 mbar (dotted black line); for higher pressure values, 10-times less precise extension channel was used. Each data point was obtained from four successive measurements of dowser domain motion, which was conditioned by the midplane flow velocity in the channel (for the B and B^* states), or from the motion of a disclination line or dust particles close to the B^* to D state transition. The selected pressure differences were repeatedly applied to no-flow equilibrium state, and the flow velocity was determined within 1 min long intervals, starting 10 s after the pressure push. Below 85 mbar, the flow velocity gradually increased up to ≈ 70 µms^{-1}, and only B state was identified. Then, the flow velocity underwent a jump (black arrow) that is most probably related to the transition to D state in a part of the channel (closer to the inlet), though we have observed chiral B^* states in the middle of the channel for several tens of seconds. Above ≈ 125 mbar, only D state was observed after 10 s time shift.

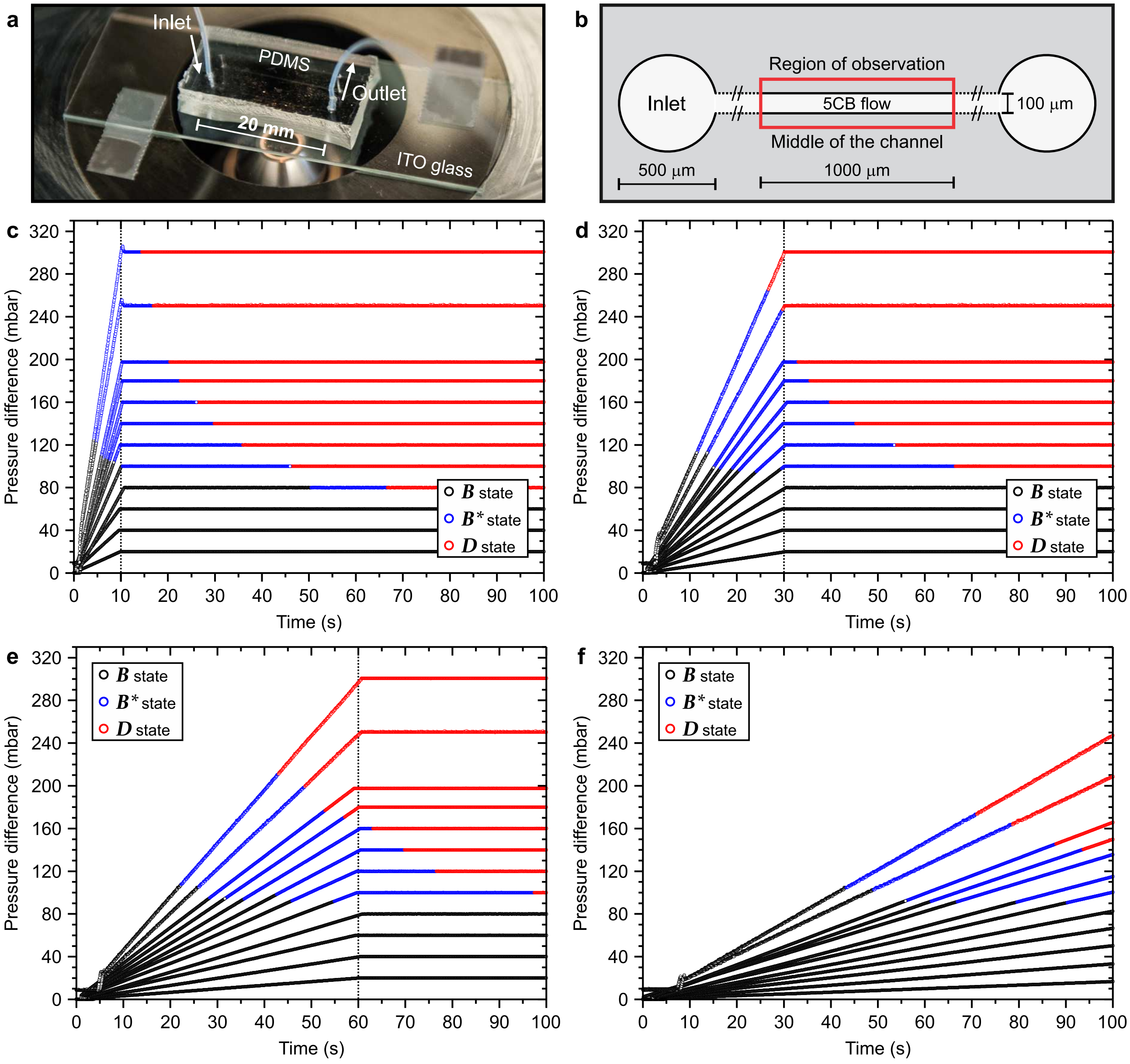

Supplementary Figure 2: **Flow pattern evolution at four different flow acceleration rates.** In these experiments, the terminal pressure differences were not attained instantly but gradually over different time intervals (dotted black lines). The pressure increase as a function of ramp time was monitored and recorded by the pressure control system, while the flow regimes (B, B^*, and D state) were identified from simultaneously recorded videos of the nematic flow. The region of observation (optical window) was set to the middle of the channel and reflected only 5 % of the channel length due to microscopy limitations. At all the acceleration rates, one can observe the prevailing B state up to ≈ 80 mbar pressure difference over ≈ 1 min time span, while the evolution and stability of the B^* and D flow states significantly depend on the applied pressure ramp. **a** A photograph of 20 mm long microfluidic channel. **b** Schematic representation of the channel with relevant length scales. **c** At fastest flow acceleration rate, the lifetime of chiral B^* states is relatively short (10 to 20 s) as the states quickly transition to flow-aligned D state which is dominant at high pressure values (above 200 mbar). **d** A bit slower acceleration rate does not considerably change the flow state evolution. **e** More moderate driving pressure increase can extend the stability range of the B^* states to ≈ 30 s, and in a certain pressure range, similar is true for slower ramps, presented in **f**. The results show that different flow states can be obtained and temporarily stabilized by precise driving pressure control.

Supplementary Notes

Supplementary Note 1: Flow velocimetry

The flow velocity measurements have been carried out using topological defect tracking technique that can be easily applied to confined birefringent liquids under polarized light microscope. The flow of a nematic 5CB was recorded under crossed polarizers at a rate of 30 frames per second. The stationary B and B^* flow states were now and then slightly perturbed by laser-induced nucleation of dowser (D) domains that instantly coupled with midplane fluid velocity and got advected with the flow in the channel. The essentially 2D domains of $50 \pm 10\,\mu\text{m}$ diameter were encircled with zero-charge topological defect loops and observed in bright birefringent colours, so their center of mass was systematically traced for several hundred micrometers along the flow direction. Since the domains are structurally equivalent to the flow-aligned nematic (D state), and are not stable in the fast flow regime, we additionally tracked the motion of a disclination line and occasional dust particles to characterize the flow velocity of bulk D state. The image files were analysed using a standard algorithm for tracking and trajectory analysis, available through the open source image processing package FIJI (based on ImageJ).

We have also tried to perform standard particle tracking technique, where silica particles of few micrometers in diameter were dispersed by sonication within the nematic host at a very low concentration. We used particles which had been treated for planar surface anchoring to avoid considerable distortion of the director field. The distortion of the director field around each particle extended only over length scales which are considerably smaller than the dimensions of the flow patterns. The comparing experiments gave very similar values of the flow velocity, so we decided to present only the results of the defect tracking method in Supplementary Fig. 1.

Since we have been using pressure control system (OB1 MK3, Elveflow), we were able to stabilize the observed flow states over 300 mbar wide pressure range. Most of the measurements have been obtained with highly precise $[0 \ldots 200]$ mbar channel. Tracking defect velocity does not provide direct measurements of the volume flow rate, which is typical for gear pump experiments – relation between the two depends also on the vertical velocity profile. The results can nevertheless be interpreted as a qualitative measure of effective viscosity. We also note that stationary flow states along the whole channel length were obtained for the B and D states only, while for the B^* states we were able to control merely few millimetres long domains which were eventually overpowered by terminal D state, so the flow velocity measurement for the B^* may include the rheological effects of both B^* and D states.
