# Supplementary material for: Microfluidic control over topological states in channel-confined nematic flows
Source: Nat Commun. 2020 Jan 2;11:59. doi: 10.1038/s41467-019-13789-9 (PMC6940393; doi:10.1038/s41467-019-13789-9)
Supplement: Supplementary file 3 — Description of Additional Supplementary Files [file 41467_2019_13789_MOESM3_ESM.pdf]

Description of Additional Supplementary Files:

Supplementary Movie 1: **Orientational transitions in a pressure-driven nematic flow.** In a weak flow, the uniformly aligned homeotropic nematic (black colour) is bowed toward the flow direction (grey-to-orange-to-reddish birefringent colours). With an increasing flow velocity the nematic undergoes a continuous symmetry-breaking transition into a chiral state of left- and right-handed domains (blue-greenish colour) that flow separated by a soliton-like structure between them. Eventually, the chiral state is replaced by a flow-aligned state (green colour) whose interface is distinguished by a disclination line. Terminal flow velocity is close to $110\,\mu\mathrm{m s}^{-1}$. Recorded under crossed polarizers at 25 fps. The channel width is 100 µm and the channel depth is 12 µm.

Supplementary Movie 2: **Symmetric formation of two oppositely-twisted chiral domains in an accelerating nematic flow.** The orientational transition can also start from the sides of the channel and propagate to the middle, where growing chiral domains collide in a flexible soliton that is prone to long-wavelength undulations. The distinct orientational order is reflected in different birefringent colours. The flow velocity is close to $90\,\mu\mathrm{m s}^{-1}$. Recorded under crossed polarizers at 25 fps. The view field size is $450\,\mu\mathrm{m} \times 150\,\mu\mathrm{m}$.

Supplementary Movie 3: **Formation of chiral flow state in weakly chiral nematic.** Gradual continuous transition from bowed to chiral nematic state occurs at relatively low flow velocity and typically starts from the middle of the channel. A small amount of a chiral dopant triggers growth of a single chiral domain with no soliton. Terminal flow velocity is close to $80\,\mu\mathrm{m s}^{-1}$. Recorded under crossed polarizers at 25 fps. The view field size is $450\,\mu\mathrm{m} \times 120\,\mu\mathrm{m}$.
